# Disparities in cancer clinical trials among low‐ and middle‐income countries: A 20‐year analysis

**DOI:** 10.1002/cncr.70067

**Published:** 2025-10-20

**Authors:** Fanny G. A. Cascelli, Milene C. Mitsuyuki, Gustavo Werutsky, Carlos H. E. Barrios, Malu V. R. Barbosa, Michelle S. Almeida, Max S. Mano

**Affiliations:** ^1^ Latin American Cooperative Oncology Group (LACOG) Brazil; ^2^ Hospital Israelita Albert Einstein Brazil

**Keywords:** cancer, clinical trials, low‐ and middle‐income countries

## Abstract

**Background:**

There are suspected disparities in clinical research (CR) development among low‐ and middle‐income countries (LMICs). This study investigated differences in number and complexity of clinical trials (CTs) and how economic growth (EG) might contribute to these disparities.

**Methods:**

For countries classified as LMICs in 2000, number, proportion of phase 1‐2/3 and independent/pharma‐sponsored CTs were documented. For correlations with EG, correlation coefficients (CC) were produced, indicating very weak, weak, moderate, strong, and very strong correlation.

**Results:**

A total of 16,977 CTs were identified. Asian countries China and South Korea experienced strong EG and increases in CTs (very strong CC). South/Southeast Asian countries had strong EG but modest increases in CTs (variable CC). Most East European countries and West Asian/Southeast European Turkey experienced robust EG and increases in CTs (moderate to strong and very strong CC, respectively). South/North American Argentina, Brazil, and Mexico had inconsistent EG but increases in CTs (weak to moderate CC). Among African countries, Egypt showed strong EG with a corresponding increase in CTs (strong CC), whereas South Africa had a weak CC. Most LMICs, except for China and South Korea, relied heavily on pharma‐sponsored CTs, with a persistently low proportion of early‐phase (1‐2) compared to late‐phase (3) CTs.

**Conclusion:**

CR development has been unequal among LMICs. Strong EG could be a contributing factor but only to some extent. Only China and South Korea meaningfully developed independent and high–complexity CR. These data reinforce the need for initiatives to support cancer research in LMICs.

## Abstract 2

This study reveals significant disparities in clinical research (CR) development among low‐ and middle‐income countries. Findings indicate that economic growth only partially contributes to CR development, with only China and South Korea significantly advancing independent and high‐complexity CR during the study period.

## INTRODUCTION

Over the next decades, most of the increase in global cancer burden will take place in low‐ and middle‐income countries (LMICs)—with rates as high as 400% in low‐ and 168% in middle‐compared to only 53% in high‐income countries (HICs).^1^ These increases in cancer incidence are attributable to various factors such as rising populations and life expectancy, growing urbanization, and lifestyle changes.^1^

Efforts to improve cancer control in LMICs should aim to reduce exposure to common modifiable risk factors such as tobacco, alcohol, and obesity but, because modifiable cancer risks play a limited role in cancer incidence and mortality, improving access to treatments and quality of cancer care in general is a priority for these nations.^2^ Access to standard of care treatments, in particular, faces unique challenges in LMICs because of soaring costs and deficient infrastructures. Although social and political actions can leverage public policies that promote early diagnosis and better treatments, this grim outlook can only be sustainably changed by the development of high‐quality local research.

Although both the volume and quality of cancer research (CR) have improved significantly globally over the last decades—this way contributing to accelerate drug development pipelines and to decrease cancer mortality—these effects have mainly been felt in HICs. Therefore, it is crucially important that LMICs continue to build their CR capacity, either by joining global studies or, most importantly, by developing their own studies and drug development programs—because their results are much more likely to pay off in terms of a wider accessibility and of addressing questions that are pertinent to each country’s reality.

Despite decades of efforts for a greater internationalization of clinical trials (CT)—with meaningful contributions from LMICs not only in terms of number of recruited patients but also of racial and cultural diversity—the reality is that they remain disproportionately concentrated in HICs. Additionally, they align poorly with the current reality of global cancer burden and are increasingly focused on interventions that provide small absolute gains to highly select groups of clinical trials that may be unrealistic (or at least not a priority) for LMICs.^3^, ^4^, ^5^

Furthermore, LMICs have historically contributed only to the very last stage of drug development trials—mostly by participating in pharma‐sponsored registration CT. These trials are usually large, global, and randomized, with investigators from LMICs barely having any role in the research design and conduction, and few opportunities to be main or senior authors; additionally, it remains unclear what and how much benefit this type of research will bring to their societies because the investigational agents, if successful, will be hardly accessible in their realities.^6^, ^7^ In this study, considering a high proportion of phase 1‐2 relative to phase 3 and independent relative to pharma‐sponsored studies as indicatives of quality and complexity of CR, we performed additional analyses to investigate this point.

Despite these challenges, LMICs have apparently managed to increase the number of cancer CTs over the last few decades. The co‐primary objectives of this study were to investigate disparities in number of cancer CTs among LMICs in terms of temporal changes and according to changes in gross domestic product (GDP) per capita. Secondary objectives were to evaluate quality and complexity of research in terms of temporal changes in proportion of early (1‐2) versus late (3) phase and independent versus pharma‐sponsored CTs.

## MATERIALS AND METHODS

First, from the World Bank website, we searched for countries from all continents and subcontinents (North and South America, Eastern Europe, South and Southeast Asia, Africa, West Asia/Southeast Europe, and East Asia) that were classified as LMICs in 2000 (the year before the ClinicalTrials.gov database became available). We employed standard international definitions (as defined by the World Bank) for classifying countries’ economies into low income, lower‐middle income, upper‐middle income, upper‐income, lower‐income, and upper‐middle income according to gross national income per capita. Country selection criteria were based on population size, economy size, and geopolitical importance, as perceived by the authors.

To collect data on CTs, we used search entries available from ClinicalTrial.gov, which is considered the most comprehensive catalogue of CTs worldwide and has been collecting data since 2000.^8^ Search methodology was as follows: we selected “advanced research”, then entered the “name of each country” in the field “location > country”. In “condition or disease”, we entered the word “cancer”; in “study type” “interventional studies (clinical trials)” and in “study start” the period of interest (i.e., every 10 years from 2001 until 2020). Subsequently, we searched the total number of cancer CTs by country, phase of the study (1, 2, vs. 3) and type of sponsor (pharma industry vs. other). The search entry “clinical trial status” was not considered.

We used the study start date in the advanced search field of ClinicalTrials.gov to identify the National Clinical Trial (NCT) number for each study and avoid counting the same study more than once.

To address the primary objective, we documented the number of CTs in selected countries that, in the early 2000s, qualified as LMICs (“low income,” “lower‐middle income,” and “upper‐middle income”) and evaluated changes in these numbers over time. We used a similar methodology to correlate these changes with GDP per capita growth. We applied similar principles for the analysis of the secondary objectives.

## STATISTICAL ANALYSES

The R software was used to perform the analyses. The strength of correlation between the number of CTs and GDP per capita was assessed using Pearson's correlation coefficient (CCs), which is an absolute number that can be negative or positive, defined as very weak (0–0.19), weak (0.2–0.39), moderate (0.4–0.69), strong (0.7–0.89), and very strong (0.9 to 1.0).

## RESULTS

Between 2001 and 2020, a total of 16,977 cancer CTs with participation of these LMICs were registered in ClinicalTrials.gov. Table 1 depicts the number of trials in each country over 5‐year periods. Most countries were able to continuously increase the number of CTs. Asian countries performed well, with China having the highest growth but robust performance also observed in South Korea. To a lesser extent, growth was also seen in East European countries. In African countries, only Egypt showed sustained growth, with stagnation and later decline observed in South Africa. In the Americas, sustained growth was mainly documented in Argentina, Brazil, and Chile, with Mexico showing a similar trend. In South and Southeast Asian countries, limited growth in CTs was seen, except perhaps for Thailand, especially in the 2001–2010 period. West Asian/Southeast European Turkey also experienced significant growth in number of CTs.

**TABLE 1:** Number of clinical trials in each country over 5‐year periods.

Region	Country	2001–2005	2006–2010	2011–2015	2016–2020	Total
Africa	Egypt	23	40	58	148	269
	South Africa	74	110	105	81	370
Asia	China	71	510	1272	3432	5285
	Republic of Korea	115	627	885	1059	2686
Eastern Europe	Czech Republic	75	237	356	374	1042
	Romania	45	168	184	159	556
	Russian Federal	113	310	419	486	1328
	Ukraine	37	112	148	199	496
West Asian/Southeast Europe	Turkey	47	109	195	277	628
North America	Mexico	65	167	182	204	618
South America	Argentina	79	176	174	218	647
	Brazil	89	254	288	369	1000
	Chile	39	77	82	150	348
	Colombia	19	51	70	69	209
	Peru	50	90	69	74	283
Southeast Asia	India	54	216	110	126	506
	Indonesia	6	21	12	7	46
	Philippines	17	62	39	37	155
	Thailand	33	118	142	146	439
	Vietnam	2	3	15	46	66
Total		1053	3458	4805	7661	16977

Next, we evaluated growth in the number of CTs as a function of changes in GDP per capita. Figure 1 depicts graphical information on growth in number of CTs according to GDP per capita per year in USD (constant 2017 international $) for each country. For this analysis, we employed previously described CCs.

FIGURE 1Growth in number of clinical trials according to gross domestic product per capita per year in USD (constant 2017 international $) for each country. GDP indicates gross domestic product; PPP: purchasing power parity; USD, United States dollar.

**Figure:**
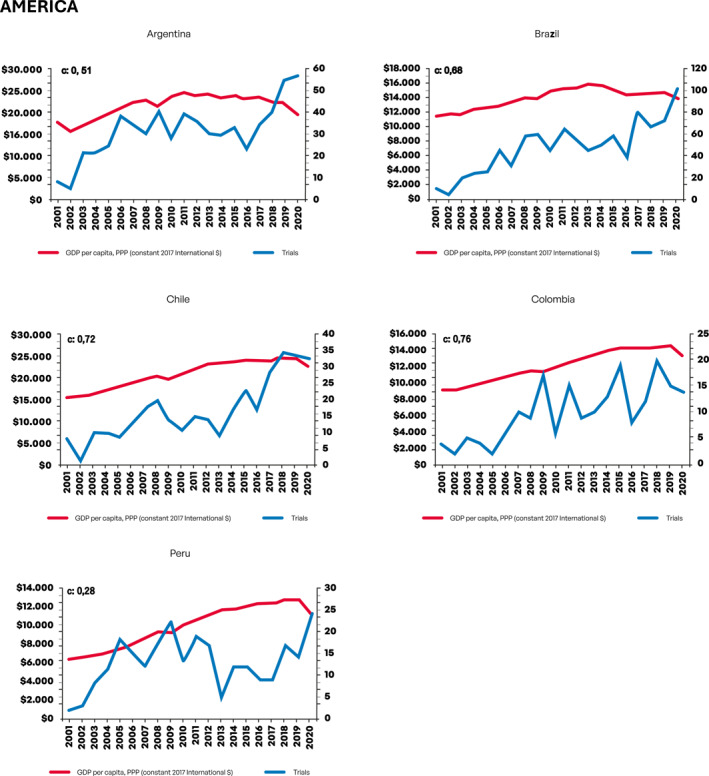

**Figure:**
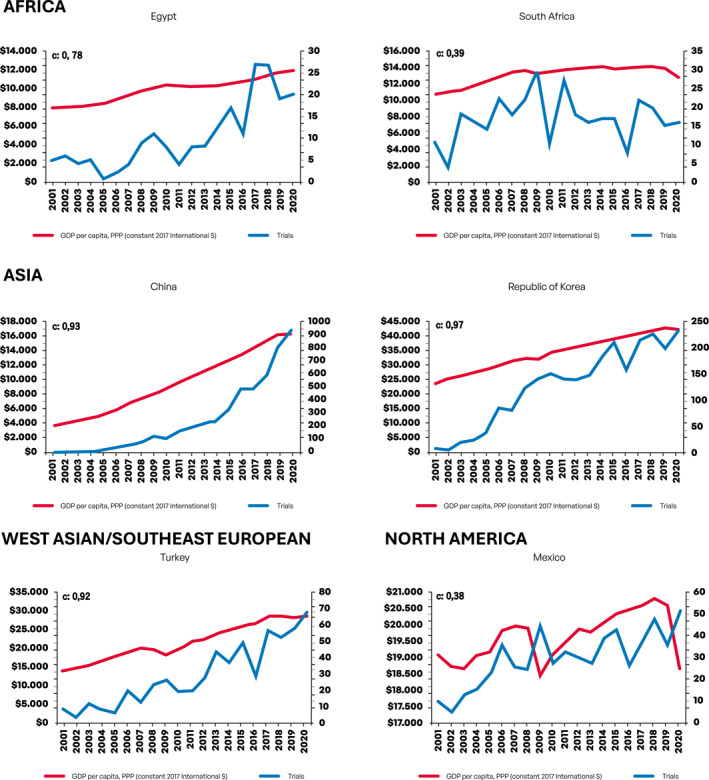

**Figure:**
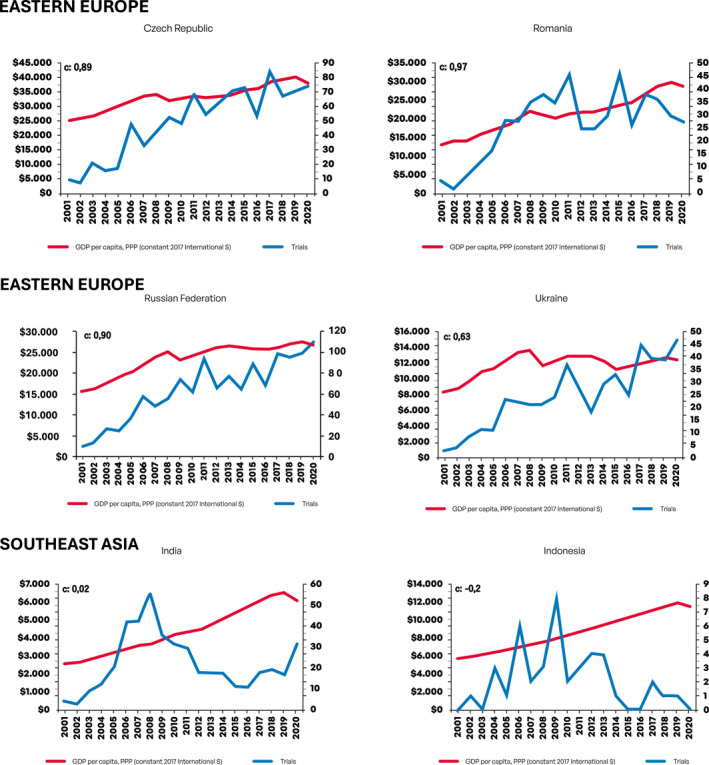

**Figure:**
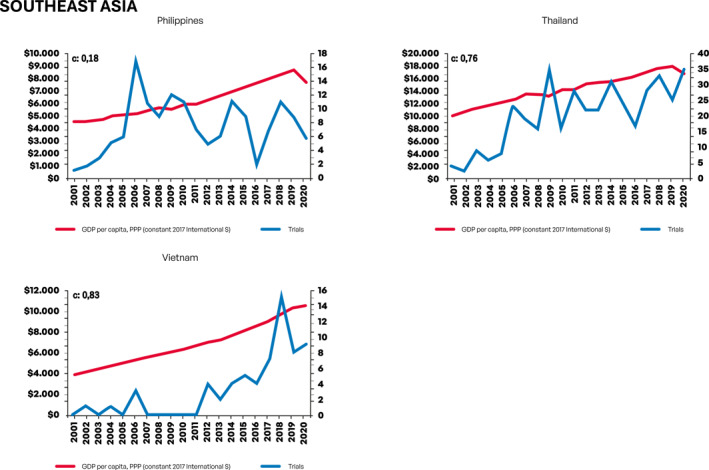

Notably, both Asian countries China and South Korea presented strong economic growth (EG) in the period and there was a very strong correlation (0.93 and 0.97, respectively) between GDP per capita and CTs growth. For African countries, EG was much more modest and only for Egypt a strong correlation (0.78) between GDP per capita and CTs growth was seen. For East European countries, robust EG was observed in Czech Republic, Romania, and Russia, with strong to very strong correlation between GDP per capita and CTs growth also observed in these countries (0.89, 0.97, and 0.90, respectively). Ukraine, conversely, had limited EG, yet a significant increase in number of CTs was seen (modest correlation, 0.63). Among South American countries, GDP per capita has largely stagnated for the two largest economies (Argentina and Brazil), with more consistent EG growth observed in Chile, Colombia, and Peru. Overall, South American countries had lower scores for the correlation between GDP per capita and CTs growth compared, for instance, with Asian countries—although a strong correlation was still evident for Chile (0.72) and Colombia (0.76). A weak correlation score was observed for Peru (0.28), which had consistent EG in the period, but with CTs only starting to grow consistently from 2013. For South and Southeast Asian countries, EG was generally strong; however, only for Thailand (0.76) and Vietnam (0.83) a strong correlation between GDP per capita and CTs growth was observed—although Vietnam had a low absolute number of CTs making this finding difficult to interpret for this country. For North American Mexico, EG was inconsistent, yet an increase in number of CTs was observed—thus resulting in a low correlation score between EG and CTs growth (0.38). For West Asian/Southeast European Turkey, both EG and CTs growth were strong, resulting in a very strong correlation between GDP per capita and CTs growth (0.92). Table 2 depicts CCs and the strength of correlation for each country.

**TABLE 2:** Correlation coefficients between economic growth and growth in number of clinical trials per country.

Country	Coefficient value (+ or –)	Strength of correlation^a^
Argentina	0.51	Moderate correlation
Brazil	0.68	Moderate correlation
Chile	0.72	Strong correlation
Colombia	0.76	Strong correlation
Peru	0.28	Weak correlation
Mexico	0.38	Weak correlation
Egypt	0.78	Strong correlation
South Africa	0.39	Weak correlation
China	0.93	Very strong correlation
Republic of Korea	0.97	Very strong correlation
Turkey	0.92	Very strong correlation
Czech Republic	0.89	Strong correlation
Romania	0.97	Very strong correlation
Russian Federation	0.9	Very strong correlation
Ukraine	0.63	Moderate correlation
India	0.02	Very weak correlation
Indonesia	–0.2	Very weak correlation
Thailand	0.76	Strong correlation
Vietnam	0.83	Strong correlation
Philippines	0.18	Very weak correlation

^a^ Interpretation: 0–0.19 = very weak correlation; 0.2–0.39 = weak correlation; 0.4–0.69 = moderate correlation; 0.7–0.89 = strong correlation; and 0.9–1.0 = very strong correlation.

**FIGURE 2:**
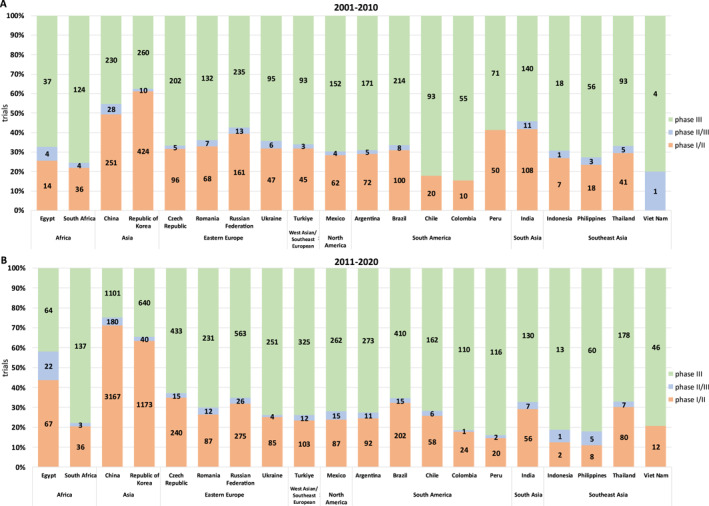
Temporal changes in the proportion of phase 1‐2 versus 3 clinical trials in low‐ and middle‐income countries. (A) 2001–2010 period. (B) 2011–2020 period.

We further evaluated temporal (over 10‐year periods, 2001–2010 vs. 2011–2020) changes in the proportion of phase 1‐2 versus 3 CTs as estimates of complexity of CR. As depicted in Figure 2A,B, most LMICs had a predominance of phase 3 CTs. China was the country with the highest growth (from 251 to 3.167) in number of phase 1/2 studies. Compared to other LMICs, both China and South Korea excelled in developing this type of CR. In the second time‐period, both these countries already had a predominance of phase 1‐2 over phase 3 CTs. Most other LMICs continued to show a low proportion of phase 1‐2 relative to phase 3 CTs, with no evidence of this scenario changing over time—an exception being Egypt, which significantly increased the proportion of phase 1‐2 relative to phase 3 CTs.

Finally, we evaluated temporal (over 10‐year periods, 2001–2010 vs. 2011–2020) changes in source of funding of CTs as an estimate of both complexity and ultimate purpose of CR. As seen in Table 3, the proportion of pharma‐sponsored trials in China fell from 41% in the 2001–2010 period to 33% in the 2011–2020 period, whereas those funded by “other organizations” (assumed to include mostly public funding) increased by six percentage points. However, a different trend was observed in South Korea, which experienced an increase in the proportion of pharma‐sponsored trials from 51% to 66%. Notably, the proportion of pharma‐funded trials remained high among South American, South and Southeast Asian, North American (Mexico), West Asian/Southeast European Turkey, and East European countries. In African countries, a similar trend was observed for South Africa but not for Egypt—that experienced a decrease in the proposition of pharma‐funded studies from 51% to 13%.

**TABLE 3:** Temporal changes in source of funding for clinical trials.

Region	Country	2001–2010			2011–2020		
		Industry (%)	Other (%)	Industry/other (%)	Industry (%)	Other (%)	Industry/other (%)
Africa	Egypt	51	36	13	13	86	1
	South Africa	85	7	8	90	3	7
Asia	China	41	51	7	33	57	11
	Republic of Korea	51	40	9	66	26	8
Eastern Europe	Czech Republic	86	8	5	88	5	7
	Romania	88	5	7	91	2	7
	Russian Federation	94	2	4	87	7	6
	Ukraine	96	0	4	92	1	7
West Asian/Southeast European	Turkey	84	12	4	92	3	5
North America	Mexico	83	11	6	83	10	7
South America	Argentina	88	3	8	93	1	6
	Brazil	80	13	7	76	15	9
	Chile	85	8	7	94	1	5
	Colombia	91	5	5	87	4	9
	Peru	88	7	5	91	1	8
South Asia	India	80	18	3	75	18	7
Southeast Asia	Indonesia	65	19	15	63	25	13
	Philippines	84	5	10	90	4	5
	Thailand	78	6	15	88	7	5
	Vietnam	40	60	0	88	7	5

## DISCUSSION

Clinical research is one of the engines of progress in health care. However, its globalization process has been problematic in many aspects. For instance, pharma‐sponsored global CTs are designed to address questions that are pertinent or realistic mainly to HICs—that are the main commercial targets of their products. Although LMICs derive unquestionable benefits from pharma‐sponsored CTs,^6^, ^7^ taking the next steps—such as conducting more complex and independently‐funded CTs (that could more properly address local needs)—has been challenging and potentially unequal among LMICs. To the best of our knowledge, this study is the first to address the question of unequal CR development among LMICs in terms of volume and quality of CR and the potential impact of EG on CR development.

Our data show that Asian countries China and South Korea excelled in terms of both EG and CTs growth. For these countries, a strong correlation between EG and CTs growth (c = 0.93 and 0.97, respectively) was seen. Similar trends, although less impressive, were observed in East European countries and East Asian/Southern European Turkey. Conversely, South and Southeast Asian countries like India, Thailand, and Vietnam—that also experienced strong EG–had mainly inconsistent growth in CTs resulting in lower correlation scores (c = 0.02, 0.76, and 0.83, respectively). Furthermore, North and South American largest economies (Argentina, Brazil, and Mexico) were also able to increase their number of CTs despite their relative economic stagnation. Although less strikingly than for China and South Korea, correlation scores were still in the high range for Chile and Colombia (c = 0.72 and 0.76, respectively) but not for Argentina, Brazil, and Mexico (c = 0.51, 0.68, and 0.38, respectively). Taken together, these data suggest that EG is a contributor but not the single determinant of CTs growth among LMICs.

What other factors might be contributing to CR growth and development in LMICs is therefore a matter of great interest. In China, for instance, a landmark reform took place in August 2015 that implemented a new evaluation process and priority approvals for CR.^9^ Additionally, in 2018, the requirement for multi‐regional CTs with drugs from other countries changed from an “approval system” to a “registration system” meaning that trials could be almost immediately started in China without additional administrative steps. This measure aimed to reduce regulatory complexity and time and appears to have been highly successful.^9^

Similarly, South Korea also underwent regulatory reforms in 2000 with the adoption of International Council for Harmonisation of Technical Requirements for Pharmaceuticals for Human Use—Good Clinical Practice followed by the introduction of the “clinical trial authorization” in 2002. Since 2004, there have been further governmental investments to improve capacity to conduct clinical trials through initiatives such as the Korea National Enterprise for Clinical Trials program.^10^

In Southeast Asia, Thailand—the country with the highest number of cancer CTs in the region by 2020—a collaborative network in cancer studies named the “Thai Society of Clinical Oncology” was created in 2018 that launched a collaborative network of medical oncologists focusing on three main types of cancer (breast, lung, and gastrointestinal cancer). This network intended to provide support, funding opportunities, and personnel for the studies, helping to boost Thai collaborative oncology research.^11^

In Turkey, between 2001 and 2020, there was a predominance of investment in research from pharmaceutical companies; according to a statement of the Association of Research Based Pharmaceutical Companies in 2012, pharmaceutical companies had invested $44 million in clinical research in Turkey. Between 2006 and 2010, for instance, of 209 drug studies submitted by the Istanbul Faculty of Medicine to the Istanbul University Ethical Committee, 174 were pharma‐sponsored.^12^

In South America, considering the data from the World Bank, Brazil was the country that by 2020 had the highest value of investment in research compared to other Latin American countries,^13^ and Colombia was the one that most increased the value of research and development expenditure per capita from 2011 to 2020. Therefore, it is possible that the unequal growth in CR between countries is due not only to GDP growth but, at least partly, also to the share of GDP invested in research and development. In 2020, for instance, 2020 Brazil invested only 1.14% whereas China invested 2.4% of their GDP in research and development.^7^, ^13^, ^14^

One of the building blocks of CR development in South America has been the creation and growth of collaborative research groups such as the Latin American Cooperative Oncology Group, which provides support for the conduction of independent research in the region through biostatical support, writing assistance, regulatory guidance, and fund‐hunting support.^15^ Another example occurred in Chile, which in 1998 founded the Grupo Oncológico Coperativo Chileno de Investigación, which is a cooperative research group that promotes collaboration between Chilean cancer centers (https://gocchi.org). These two groups have supported many studies, mostly pharma‐sponsored but also a growing number of independent studies.^15^

The African continent produces only 2% of the world research output despite accounting for 15% of the global population.^16^, ^17^ South Africa, as shown in our research, presented a predominance of phase 3 studies and had a decrease in number of CTs between 2001 and 2020. One of the reasons for this may be the flight of trained professionals to HICs because of lack of financial and other incentives. In Africa, in general, there are few phase 1‐2 studies partly because there are scarce resources for developing translational research that make these studies feasible.^2^ Conversely, Egypt showed more progress than many other countries—evolving from a predominance of phase 3 studies in the 2001–2010 period to a predominated of phase 1‐2 studies in the 2011–2020 period. This change may have occurred for various reasons such as a fast‐growing, largely uninsured and treatment‐naive population, large pool of diseases within the population, attractive research infrastructure, lower costs for conducting CTs, and lack of official national regulations on clinical trials that likely reduces bureaucracy burden.^18^

We addressed points that we defined as “CTs complexity,” for instance, the proportion of early phase (1‐2) relative to late‐phase (3) CTs. It is well known that, because early‐phase CTs require lower numbers of patients and are more difficult to perform because they also require more technology and infrastructure, they have been mainly conducted in high‐complexity units in HICs. Conversely, large phase 3 registration studies tend to be global studies involving significant recruitment from LMICs. Interestingly, we showed that only China and South Korea had a meaningful proportion of phase 1‐2 relative to phase 3 CTs and both have managed to increase this proportion to the point of making them the predominant type of CTs in the second time period (2010–2020). Notably, over this same period, these countries appear to have made significant investments in home‐grown pharmaceutical industry^10^ in addition to significant reforms in their regulatory systems^10^ and have also set up numerous well‐structured research facilities^9^—facts that could themselves account for these progresses.^9^, ^10^ Conversely, except for Egypt, most other LMICs showed virtually no progress in terms of increasing the proportion of phase 1‐2 relative to phase 3 CTs over this period.

Too much reliance on pharma‐sponsors CR is another matter of concern because, as previously addressed, these trials most often fail to address the actual needs of LMICs’ populations and health systems.^19^ In this aspect, only China (and smaller Egypt) made significant progresses in increasing the proportion of independent relative to pharma‐sponsored CTs. Assuming that “other” in ClinicalTrials.gov means “mainly public or institutionally funded” research, this is a tremendous achievement in terms of addressing China and Egypt populations’ needs and promoting their CR internationally with more first and senior authorship opportunities for their researchers.

The fact that some countries with strong EG failed to meaningfully develop their CR deserves consideration. Data from this study do not address the potential causes for this discrepancy; however, we speculate that obstacles to CR development are varied and not always related to financial support, including, for instance, to what extent science is valued by countries as an engine to achieve and maintain EG, and cultural aspects such as physicians’ and patients’ perceptions on clinical trials (e.g., in a recent survey, patients with cancer from Brazil had a worrisome perception of serving as “guinea pigs“ in clinical trials).^20^ Many specific actions from countries addressed in previous paragraphs, especially in terms of their capacity and willingness to implement regulatory reforms, better financial compensation for researchers that at least match that achievable with routine patient care, staff training, and so on might be achievable without the need for heavy financial investments. In Table 4, we suggest potential actions that could foster the development of CR in LMICs, some of them with modest or virtually no financial investment. Advice for LMICs willing to foster CR has also been comprehensively addressed elsewhere by Barrios et al.^7^

**TABLE 4:** Potential actions that could foster the development of clinical research in LMICs.

Action	Level of financial investment required
Demystify participation in CTs among patients and physicians	Minimal
Regulatory reforms that might facilitate swift and inexpensive research approval	Minimal to modest
Fair financial compensation for research teams that at least matches that achieved with routine clinical practice	Variable (potentially modest to high)
Access to physician and research staff training	Minimal to modest
Availability of funding for independent research	Variable (potentially modest to high)
Creation of collaborative research groups to support CR	Variable (potentially modest to high)—however, ideally, collaborative research groups should work to make themselves sustainable over time
Avoid flight of trained professionals to HICs	Variable (potentially modest to high)

Abbreviations: CR, clinical research; CTs, clinical trials; HICs, high‐income countries; LMICs, low‐ and middle‐income countries.

This study has some limitations, including the country selection process. Because it was unfeasible to analyze all countries, and our intention was to provide a snapshot of the situation rather than a comprehensive global analysis, country selection was based on authors’ perception of relative country population size, economy size, and geopolitical importance. In fact, our various attempts to establish strict criteria for country selection proved unsuccessful because of complexities related to the number of variables involved. Consequently, the number of countries included is relatively low on a global scale, which could be considered another limitation.

Potential biases related to the quality of information on ClinicalTrials.gov should be mentioned. For instance, information on sponsorship is a responsibility of the investigator and/or sponsor, so that we cannot rule out some level of bias due to misclassification. However, because of the high stakes involved with registering incorrect information, of the fact that, for pharma‐sponsored CTs, the information is usually entered by the sponsors themselves who are usually international companies that follow strict compliance regulations, and of the large number of CTs included in this study, we consider this unlikely to affect our results. We also raise the possibility of underreporting of CTs in certain regions; although ClinicalTrials.gov is the most comprehensive and renowned CTs registry, the existence of other registries that could compete with ClinicalTrials.gov, such as the International Clinical Trials Registry Platform and the Pan‐African Clinical Trials Registry, should be noted.^21^ Finally, ClinicalTrials.gov may have failed to comprehensively capture CTs information in its initial years, which could be another source of biases.

In this study, low‐ (only two countries in 2000) and middle‐income countries were analyzed together as a group. This is because these countries share many characteristics, and most scientific works to date have taken this same approach. However, we cannot rule out that some differences between low‐ and middle‐income countries could affect their capacity and interest in developing clinical research, thus representing another potential bias in this study.

Finally, we also acknowledge that we had to conduct complex (and potentially imperfect) mathematical equations to establish the correlation coefficients used in this study.

As expected, during the study period (2001–2020), some countries (such as South Korea) experienced significant economic development and started being classified as high income, whereas others have progressed from middle‐low to middle‐high income. This economic mobility is what allowed us to address one of the main objectives of the study, which was to correlate growth in CTs with EG.

Cancer CR is important for countries not only for the purpose of improving patients’ prognosis and quality of life, but also of promoting science and generating jobs and tax revenues—this way also contributing to their EG.^22^ Our data suggest that periods of economic woes—that are not unusual on the long road to economic development—should not be a deterrent to continued CR development. By unmasking the unequal CR development among LMICs, this study may serve as a roadmap for LMICs willing to match the top performers.

## AUTHOR CONTRIBUTIONS

**Fanny G. A. Cascelli:** Writing—original draft; project administration; data curation; conceptualization. **Milene C. Mitsuyuki:** Writing—review and editing; supervision; methodology; formal analysis. **Gustavo Werutsky:** Writing—review and editing; supervision; resources; and validation. **Carlos H. E. Barrios:** Writing—review and editing; supervision; resources; validation. **Malu V. R. Barbosa:** Writing—review and editing; validation. **Michelle S. Almeida:** Writing—review and editing; validation. **Max S. Mano:** Writing—original draft; writing—review and editing; conceptualization; supervision; methodology; validation; project administration.

## CONFLICT OF INTEREST STATEMENT

Max S. Mano reports honoraria for lectures and/or advisory boards from Roche, Novartis, Lilly, MSD, AstraZeneca, Daiichi‐Sankyo, and Gylead; and travel support from Gilead. Carlos H. E. Barrios reports consulting fees from Novartis, Pfizer, Roche/Genentech, MSD, Astra Zeneca, Lilly, Daiichi, and Gilead; honoraria from lectures from Adium, Novartis, Pfizer, Roche/Genentech, MSD, Astra Zeneca, Lilly, Daiichi, and Gilead; meeting and/or travel support from Novartis, Roche/Genentech, MSD, Astra Zeneca, Lilly, Daiichi, Gilead; participation in a data monitoring committee from Roche/Genentech; and his institution received grants from Amgen, Astra Zeneca, Aveo Oncology, BioNTech, BMS, Daiichi Sankyo, Dizal Pharma, Exelixis, Fortrea, Gilead Sciences, GSK, ICON, IQVIA, Janssen, Labcorp, Lilly, Medpace, MSD, Novartis, Novocure, Nuvisan, OBI Pharma, Parexel, Pfizer, PharmaMar, PPD, PSI, Regeneron, Roche/Genentech, Samsung, Sandoz, Sanofi, Seagen, Servier, Stemline, Syneos Health, Taiho, Takeda, Tolmar, TRIO, and Worldwide. Gustavo Werutsky reports consulting fees from Merck, Daiichi Sankyo, Bristol, Roche, and Novartis; honoraria for lectures/presentations from Pfizer, AstraZeneca/MedImmune, Daiichi Sankyo, Merck, and MSD; travel expenses from AstraZeneca; and his institution received grants from Novartis, Roche/Genentech, AstraZeneca/MedImmune, Lilly, GSK, Pfizer, Bristol, Roche, MSD, Merck, Bayer, Janssen, Astellas, Libbs, and Takeda. Malu V. R. Barbosa reports honoraria for lectures from AstraZeneca and MSD; travel expenses from Amgen and MSD; and consulting fees from AstraZeneca. The other authors declare no conflicts of interest.

## Data Availability

Data were collected from public sources (ClinicalTrial.gov and World Bank databases). Search processes are detailed in Materials and Methods. Data were then transferred to an excel file from which the statistical analyses were performed. These files can be made available to readers on a (reasonably justified) request to the corresponding author. There are no additional data to be made available. We may require a signed data access agreement to preserve authorship rights.
